# Annotations

**Published:** 1903-01-31

**Authors:** 


### ANNOTATIONS.

### The Limits of an Operation.

IT is an interesting question, ethical as well as surgical, whether an operating surgeon should ever, if we may so put it, exceed his instructions? Should he, that is, limit his interference to the mere cure or relief of the trouble in regard to which he is called in, or should he at the same time set right any other matter requiring or apparently deserving of rectification which he may come across in the course of the operation? On the one hand, it may be said that by leaving untouched a thing which will probably require surgical intervention later on he is doing his patient an ill-turn, or as ill-natured folk might say is "making a job for himself," while on the other, it may very justly be held that as any extension of the operation may possibly increase its danger no such extension is justifiable. The question has recently cropped up in connection with appendicitis. If, as is now so widely held, a man who has recovered from an attack of appendicitis may properly be urged to have his appendix removed lest worse evil should befall him on the next occasion, would not a surgeon who for any reason has to open an abdomen be doing right in utilising such a golden opportunity for getting rid of this so often peccant organ? Dr. Howard Kelly has lately discussed this important question and concludes against such a proceeding where the appendix is normal on the grounds that such an extension of the operation and

ANNOTATIONS—*continued.*

of the time involved in its performance might add to its danger, and that we have no knowledge that the possession of a *normal* appendix involves any risk. When, however, the appendix is abnormal, when it contains concretions, when it is abnormally long, when it is adherent, and when it is so near the site of operation that it might become adherent, the affair is different, and the organ had better be removed. It is not only in regard to appendicitis, however, that this question arises. In operations for ovarian tumours a problem of the same nature but of greater complexity has to be faced when the opposite organ is found to be affected in a minor degree, while in cases of Cæsarian section it sometimes becomes a serious question whether or not so to extend the operation as to prevent the same mischief happening again. The only safe rule seems to be that the surgeon should not allow himself to be fettered by limitations imposed beforehand, for it will, without doubt, be best for the patient that the operator should be left with a free hand. But cases are sure to arise occasionally in which the patient or those responsible will insist on imposing limitations, and in that case these must be respected, even to the patient's detriment.

### Fish, Leprosy, and Mr. Hutchinson.

NOTWITHSTANDING the ridicule with which the *British Medical Journal* has chosen to treat the subject we cannot but express our sympathy with Mr. Jonathan Hutchinson, and our admiration for the energy and enthusiasm which have led him to visit India and Ceylon, in the hope of clearing up some of the points which militate most strongly against his hypothesis that leprosy is caused by the eating imperfectly cured fish. As need hardly be said, one of the points most frequently and effectually raised against the fish hypothesis is the prevalence of the disease in India among people who not only are far removed from the coast, but the tenets of whose religion are distinctly opposed to the eating of anything that has had life, including, of course, fish. Mr. Hutchinson, however, has but little faith in the statements of the Brahmins as to their abstinence from fish, preferring apparently to accept the Hindoo proverb quoted by Kipling in "Kim" to the effect that "where there are no eyes there is no caste." But, however this may be, one of the objects of his present mission is to ascertain how far the facts, as exemplified in India, may be taken as negativing or supporting his hypothesis. It may be well to point out that the theory so vigorously maintained by Mr. Hutchinson as to the relation between fish and leprosy has important bearings on the etiology of other infective diseases. Hansen's discovery of the lepra bacillus seems at first sight to give the *coup de grâce* to the fish hypothesis. But if Mr. Hutchinson could uphold the fact of a distinct connection between consumption of imperfectly cured fish and the prevalence of a disease which is admittedly due to bacillary infection, we might have to widen our notions considerably as to what we mean by a predisposing cause. The selective action of the different infections, or in other words the nature of "susceptibility" is still to a large extent a mystery, and if Mr. Hutchinson could show that the bacillus of leprosy cannot, or at least does not commonly, develop except in the presence of toxins derived from such a dietetic error as the consumption of decomposing fish, some light might be thrown upon many of the strange freaks shown by infectious disorders in the selection of their victims.

### "Malt" and other Whiskies.

WHY the papers should just now devote so much attention to the subject of whisky is perhaps not very apparent, but in view of the many conflicting statements which have been made as to the comparative merits of different kinds of whisky, it may be well to say a word as to the position which medical practitioners may fairly take up in any discussion on the subject. With us the sole question is, not as to whether the whisky in question is "malt," or "grain," or "blend," or what not, but whether it is as innocuous as whisky can be; and in deciding this question we have to remember, first of all, how ignorant we are as to the exact nature of the substances which give to raw spirits their injurious qualities, and as to the changes which take place while such raw spirit matures into a mellow and full-flavoured whisky; and, secondly, that, ignorant as we are as to all this, we do know that by mere keeping, this maturing process does take place; so that, given a malt origin for a fair proportion of the spirit to begin with, a "harmless" whisky will result by mere efflux of time. What we have to insist upon then is age rather than any elaborate analyses for so far as soundness is concerned (although flavour is another affair) the age of the compound is really far more important than any statement, however veracious, upon the label as to the malt from which it was brewed or the glen in which it was distilled. We know of course that many assertions are made as to the powers of modern chemistry to detect the several ingredients which exist in such varying proportions in whiskies of different ages and different origins. But our experience of the operations of the Sale of Food and Drugs Act makes us absolutely sceptical as to any effective check being placed by its means on the sophistication of whisky. Expert evidence—and in this case it would be all expert evidence—is under too dark a cloud just at present to allow us to believe that conviction could be obtained except in the very grossest cases. Let us not be led by analysts, but trust to age. *Tempus omnia vincit.* The problem may well be narrowed down to the question how to secure an assurance as to age.

### Hospital Abuse in Bradford.

MR. BENTHAM, chairman of the Bradford Board of Guardians, has been lecturing at the Bradford Liberal Club on the administration of the charities of the town. At the Children's Hospital, he said no inquiries whatever were made in the out-patient department as to the circumstances of parents, all comers being dealt with indiscriminately, while even in regard to in-patients, children were admitted without investigation. The circumstances of the parents were inquired into afterwards, and if they were found able to contribute they were expected to do so, but he was told that even as to this the parent's word was taken without further investigation.

